# Freeze-quenched maize mesophyll and bundle sheath separation uncovers bias in previous tissue-specific RNA-Seq data

**DOI:** 10.1093/jxb/erw463

**Published:** 2017-01-02

**Authors:** Alisandra K Denton, Janina Maß, Canan Külahoglu, Martin J Lercher, Andrea Bräutigam, Andreas P M Weber

**Affiliations:** 1 Institute of Plant Biochemistry, Cluster of Excellence on Plant Sciences (CEPLAS), iGRAD-Plant Program, Heinrich-Heine-University, Düsseldorf, Germany; 2 Institute of Informatics, Cluster of Excellence on Plant Sciences (CEPLAS), iGRAD-Plant Program, Heinrich-Heine University, Düsseldorf, Germany; 3 Network Analysis and Modeling Group, IPK Gatersleben, Corrensstrasse, Stadt Seeland, Germany

**Keywords:** C_4_, cell separation, maize, meta-analysis, transcriptomics

## Abstract

The high efficiency of C_4_ photosynthesis relies on spatial division of labor, classically with initial carbon fixation in the mesophyll and carbon reduction in the bundle sheath. By employing grinding and serial filtration over liquid nitrogen, we enriched C_4_ tissues along a developing leaf gradient. This method treats both C_4_ tissues in an integrity-preserving and consistent manner, while allowing complementary measurements of metabolite abundance and enzyme activity, thus providing a comprehensive data set. Meta-analysis of this and the previous studies highlights the strengths and weaknesses of different C_4_ tissue separation techniques. While the method reported here achieves the least enrichment, it is the only one that shows neither strong 3′ (degradation) bias, nor different severity of 3′ bias between samples. The meta-analysis highlighted previously unappreciated observations, such as an accumulation of evidence that aspartate aminotransferase is more mesophyll specific than expected from the current NADP-ME C_4_ cycle model, and a shift in enrichment of protein synthesis genes from bundle sheath to mesophyll during development. The full comparative dataset is available for download, and a web visualization tool (available at http://www.plant-biochemistry.hhu.de/resources.html) facilitates comparison of the the *Z. mays* bundle sheath and mesophyll studies, their consistencies and their conflicts.

## Teaser

Systems analysis of freeze-quenched mesophyll and bundle sheath tissues enriched under liquid nitrogen provides a reliable alternative to previously used separation techniques that showed bias in RNA quality.

## Introduction

Specialization and coordination between two cell types improves photosynthetic efficiency in most C_4_ photosynthetic plants. Specifically, most C_4_ plants shuttle carbon from a surrounding mesophyll (M) tissue into a surrounded bundle sheath (BS) tissue (Hatch, 1987). The shuttling concentrates CO_2_ around the carbon fixing enzyme, Rubisco, thereby suppressing photorespiration and increasing photosynthetic efficiency. This lends selective advantage to C_4_ plants in photorespiration-inducing (e.g. hot and arid) environments (Schulze *et al.*, 1996). The high photosynthetic efficiency and stress tolerance of C_4_ species has led to interest in engineering the trait. However, the complexity of the trait—with many changes to anatomy and metabolism beyond the core biochemical pump—makes this an ambitious goal, which will require a full systems-level understanding of both the mature C_4_ trait and its development to be achieved (Sage and Zhu, 2011).

BS and M cells show extensive specialization in metabolism and anatomy in C_4_ plants. In the classic C_4_ arrangement—Kranz anatomy—enlarged BS cells form a ring around the vascular bundle and are in turn surrounded by M cells (Hatch, 1987). Narrow vein spacing means each M cell borders a BS cell, allowing direct transfer of metabolites between them. Compared with a C_3_ leaf, there is a massive increase in the relative amount of BS tissue, allowing for a division of labor between cell types that includes both photosynthesis and major facets of other metabolism (Majeran *et al.*, 2010; Friso *et al.*, 2010). Following Rubisco, most enzymes in the Calvin–Benson–Bassham Cycle (CBBC) and the linked photorespiratory cycle are restricted to the BS (Broglie *et al.*, 1984; Rawsthorne *et al.*, 1988; Döring *et al.*, 2016). In *Z. mays*, distribution of photosystem II and therefore linear electron transport and reducing equivalent regeneration are restricted to the M, while the BS relies on ATP from cyclic electron transport around photosystem I and biochemical shuttles that transfer reducing equivalents to the BS for energy (Romanowska *et al.*, 2008; Wang *et al.*, 2014; Bellasio and Griffiths, 2014). Subsets of metabolism are divided up between the two cell types with, for instance, amino acid, nucleotide, and isoprenoid synthesis in the M, and sulfur metabolism and starch synthesis in the BS (Majeran *et al.*, 2005; Friso *et al.*, 2010).

Information on anatomical and metabolic changes has been gained through comparative proteomic and transcriptomic studies both between C_3_ and C_4_ species (e.g. Bräutigam *et al.*, 2011, 2014; Gowik *et al.*, 2011; Wang *et al.*, 2014; Covshoff *et al.*, 2016), and between isolated tissue types (Majeran *et al.*, 2005; Friso *et al.*, 2010; Li *et al.*, 2010; Chang *et al.*, 2012; Tausta *et al.*, 2014; John *et al.*, 2014; Aubry *et al.*, 2014). Many of the differences between cell types are set up early in development, and tissue maturation studies have obtained mechanistic insights. For instance, comparison of C_4_ and C_3_ Cleomaceae species linked delayed photosynthetic differentiation to extended vein proliferation and ultimately closer vein spacing in the C_4_ species (Külahoglu *et al.*, 2014). In *Z. mays* carefully comparing the primordia of Kranz leaf tissue with non-Kranz husk tissue implicated the recruitment of the ScareCrow regulatory module from the root epidermis to BS cells (Wang *et al.*, 2013). Potentially due to the difficulties of isolating cell types, to date there has only been one transcriptomics (Tausta *et al.*, 2014) and one proteomics (Majeran *et al.*, 2010) study that have looked at immature M and BS tissue. These studies have shown the early establishment of tissue specificity of major C_4_ enzymes and the roles of M and BS cells in sink *vs* source tissue to logically reflect the broader changes between source and sink tissue. As neither of the above studies could look at metabolites, and interstudy comparisons have produced distinct results on cell specificity—particularly of transcription factors (Tausta *et al.*, 2014)—we judged further analysis to be warranted.

Here we successfully perform an ‘omics’-scale analysis on developmental tissue separated by a method developed by Stitt and Heldt (1985), and thus simultaneously capture changes in the transcriptome, enzymatic activities, and the metabolome. A subsequent meta-analysis of this and other BS and M separation studies highlights the strengths and weaknesses of each of the various separation methods, and the advantages of using complementary techniques. The comparative dataset has been made available for visual exploration or download, and can assist both in experimental design both for BS/M related studies and for studies in the broader category of tissue separation.

## Materials and methods

### Plant genome data

Genome and gene-model data was downloaded for *Setaria viridis* (v1.1/v311; Bennetzen *et al.*, 2012) and *Panicum virgatum* (v1.1/v273; DOE-JGI, 2016) from Phytozome 11.0 (Goodstein *et al.*, 2012). The AGPv3.22 release of the *Zea mays* genome with the 5b+ filtered gene set was obtained from ensemble plants (Kersey *et al.*, 2016) and Gramene (Tello-Ruiz *et al.*, 2016), respectively. Orthologs were identified by best BLAST (Altschul *et al.*, 1997) hit from *Z. mays* to *S. viridis* or *P. virgatum.*

### External RNAseq data

Complementary RNAseq data were downloaded from the sequence read archives (Kodama *et al.*, 2012) and European nucleotide archives (Leinonen *et al.*, 2010). We included two additional *Z. mays* BS and M separation studies (Chang *et al.*, 2012: SRP009063; Tausta *et al.*, 2014: SRP035577); corresponding whole developmental leaf sections (Li *et al.*, 2010; SRP002265); *Z. mays* tissue atlas (Sekhon *et al.*, 2013; SRP010680); and primordial leaf and husk tissue (Wang *et al.*, 2013; SRP028231). The non-*Z. mays* studies were separation of BS and M cells in *S. viridis* (John *et al.*, 2014; ERA275647) and *P. virgatum* (Rao *et al.*, 2016; SRP062667).

Note that as the original authors included the same precise set of sequences for BS and M tissues in section 14 (Li *et al.*, 2010; Tausta *et al.*, 2014), and reported the same plant growth conditions, we’ve considered these studies broadly comparable. However, to avoid redundancy, the BS and M samples for section 14 are only included with Tausta *et al.* (2014).

### Plant growth conditions and harvest

*Z. mays* B73 was grown in the summer of 2012 under conditions previously described (Pick *et al.*, 2011). The third leaf was harvested when it measured 18 cm from the second ligule to the leaf tip. Two different harvesting methods were performed. In the first, a leaf gradient with five sequential developmental slices (4 cm each) was harvested with the ‘leaf guillotine’ (see Fig. S1A available at Dryad Digital Repository http://dx.doi.org/10.5061/dryad.tf6q6;Pick *et al.*, 2011). This method required 10 s to extract the third leaf and properly align it, which does not allow for reliable estimates of the high-turnover photosynthetic metabolite distributions. Therefore, a second harvesting method was performed, in which the plants were positioned above two liquid nitrogen containers and two 8 cm slices were cut with connected scissors (see Fig. S1A, B at Dryad) achieving a delay of less than 1 s between slicing and flash-freezing. Metabolite abundance and enzyme activity were measured from both harvest sets; the full five-slice gradient was used for RNAseq.

### Tissue enrichment

M and BS tissues were enriched using a method modified from Stitt and Heldt (1985). Ground material was filtered through 250, 80, and 41 μm meshes on liquid nitrogen. Three fractions were selected for further analysis. The ‘BS-e’ fraction showed the most enrichment of BS tissue (it did not pass through 80 μm mesh); the ‘M-e’ fraction showed most enrichment in M tissue (it passed through 41 μm mesh); and the ‘I-e’ fraction showed intermediate, but consistent, proportions of tissues (it did not pass through 41 μm mesh).

### Extraction and abundance measurements metabolites and enzymes

Enzymes were extracted and desalted as described in Bräutigam *et al.* (2014), and the enzyme activity was measured through colorimetric assays as described in Hatch and Mau (1977) and Walker *et al.* (1995). Metabolites were extracted and quantified via gas chromatography–electron-impact time-of-flight mass spectrometry as described in Rudolf *et al.* (2013). Both low-signal metabolites and individual replicates with a percentage abundance in BS more than 3 standard deviations from the mean were excluded. The integrated peaks were divided by the area of the ribitol (internal standard) peak and the fresh weight, and to further reduce noise and compensate for FW/DW differences between the cell types by the mean abundance for the replicate. Therefore, normalized differences between metabolites represent not absolute distribution, but distribution relative to the other metabolites, particularly sucrose and the other highly abundant metabolites.

### Sequencing and estimating transcriptional abundances

RNA was extracted with QIAGEN RNeasy Plant kits, according to the manufacturer’s instructions except for an extra wash step in 80% ethanol after the standard wash steps. Libraries were prepped from RNA with an RNA integrity number >8 and sequenced with the Illumina HiSeq 2000 platform. The quality was checked with FastQC (Andrews, 2010). Quality and adapter trimming was performed with Trimmomatic (Bolger *et al.*, 2014). Trimmed reads were mapped to their respective genomes with Tophat2 (Kim *et al.*, 2013) and the unique counts per locus were quantified with HTSeq (Anders *et al.*, 2015); transcripts per million (TPM) was calculated from the unique counts and gene length. Coverage metrics including 3′ bias were calculated with PicardTools 2.4.1: CollectRnaSeqMetrics (Wysoker *et al.*, 2012). Non-default parameters used for bioinformatics programs are provided (see Table S1 at Dryad). The same pipeline was used for all studies except as necessitated by experimental differences (e.g. paired *vs* single end reads), or otherwise noted.

### Differential expression and tissue specificity normalization

Differential expression *P*-values and log_2_ fold changes were calculated with EdgeR (Robinson *et al.*, 2009). Where no replicates were available (Chang *et al.*, 2012), the mean common dispersion from the remaining studies was used. Additionally, due to the low level of enrichment achieved in this study, ContamDE (Shen *et al.*, 2016), a cross-contamination tolerant package for RNAseq statistics, was employed for the data generated here. As necessary for interstudy comparisons in *Z. mays*, log_2_ fold changes from edgeR (Chang *et al.*, 2012; Tausta *et al.*, 2014) and ContamDE (this study) were quantile normalized, and the fully normalized TPM back calculated from the quantile normalized log_2_ fold change and mean TPM.

### Estimation of initial tissue specificity by ‘deconvolution’

The distribution of metabolites and enzyme activities was compared with the distribution of markers to estimate the original tissue specificity in a method modified from Stitt and Heldt (1985). First, all data were converted into fraction of total by developmental slice. Second, marker enzyme activities were used as proxies for the amount of M (phosphoenolpyruvate carboxylase (PEPC) activity) and BS (NADP-malic enzyme (ME) activity) tissue in each enrichment fraction. The slope of a regression line between the ln(target/M) against ln(BS/M) estimated the fraction of the target found in pure BS (see Fig. S1C at Dryad). *P*-values were calculated with a null hypothesis of slope=0.5 (50% M, 50% BS). This was automated with a linear regression in R and calculated for every metabolite and non-marker enzyme. To estimate the ‘pure’ abundance values, the estimated fraction in BS and M (1–fraction BS) were multiplied by 2× the average abundance value for the developmental slice.

### Functional category enrichment testing

Functional categories were assigned with Mercator (Lohse *et al.*, 2014). Enrichment was tested with Fisher’s exact test, and the false discovery rate calculated according to (Benjamini and Yekutieli, 2001).

### Statistics

Unless otherwise noted, all statistical analysis was performed in the R statistical environment (R Development Core Team, 2011) and whenever a test was performed more than 20 times, the false discovery rate (Benjamini and Hochberg, 1995) was calculated from the resulting *P*-values.

### Accession numbers

The reads related to this article have been deposited in the Sequence Read Archives under the accession number SRP052802.

## Results

### Validation of separation method

Here, we enriched BS and M cells along a developing *Z. mays* leaf by grinding and serial filtration (Stitt and Heldt, 1985). Two harvesting methods were used, the first using a ‘guillotine’ (Pick *et al.*, 2011) to sample five contiguous 4 cm slices from tissue just emerging from the ligule (slice 5) to the leaf tip (slice 1). In the second, targeted at capturing unadulterated metabolite levels, two 8 cm slices were harvested in full illumination and quenched in liquid nitrogen within a second of cutting. M and BS tissues were enriched using a method modified from Stitt and Heldt (1985) that capitalizes on the distinct physical properties of M and BS cells to enrich them in different separation fractions as ground tissue is filtered through serially smaller meshes over liquid nitrogen. The activity of C_4_ enzymes and the metabolite levels were measured from both harvests, and RNAseq was performed on material from the five-slice gradient.

The distribution of tissue specific markers indicated BS and M tissue were successfully enriched (see Fig. S1D and Dataset S1 at Dryad). The classic BS marker is NADP-ME, the enzyme responsible for releasing the carbon from C_4_ acids in the BS. NADP-ME activity and transcripts were both higher in the coarsest (from here on, BS-e for bundle sheath enriched) separation fraction; in between in the middle (from here on, I-e for intermediate enrichment) separation fraction; and lowest in the finest (from here on, M-e, for mesophyll enriched) fraction (see ‘Materials and methods’ for details). The classic M marker, PEPC, the C_4_ fixing enzyme, showed the opposite pattern, with highest activity and transcript abundance in the fine, M-e fraction. While the enrichment was strongest in mature tissue, it was also apparent in the youngest tissue (slice 5). For non-marker enzymes and metabolites, the original distribution was estimated based on the marker enzymes (see ‘Materials and methods’; Fig S1C at Dryad).

**Fig. 1.**
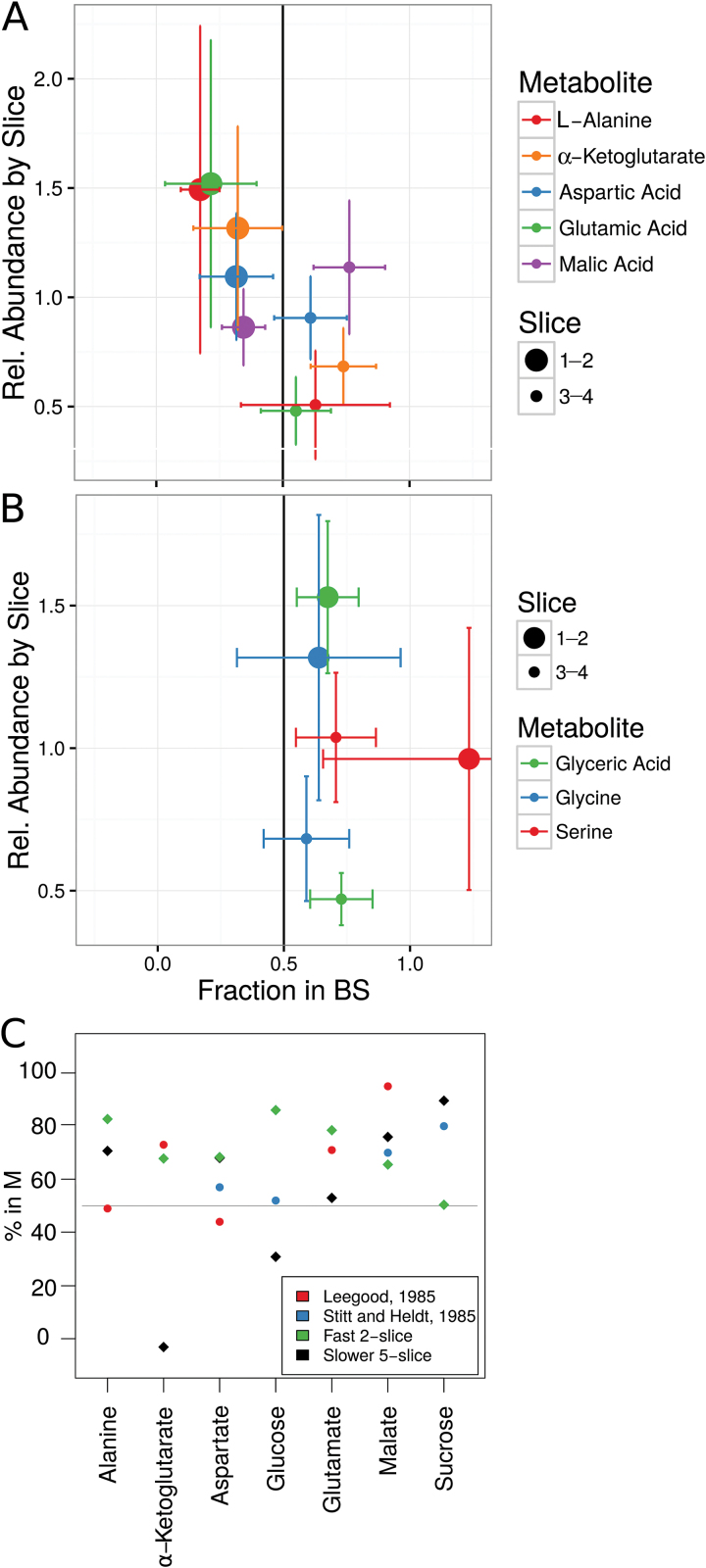
Metabolites. (A, B) The estimated tissue enrichment and abundance of measurable metabolites associated with the photorespiratory cycle (A) and the C_4_ cycle (B). Error bars indicate standard error. (C) Comparison of metabolite tissue enrichment measured by Leegood (1985) and Stitt and Heldt (1985) with the average of slice 1 and 2 in the slower five-slice harvest and slice 1–2 in the faster two-slice harvest.

This enrichment method was chosen over other separation methods both for sample integrity and to obtain data on metabolite abundance, enzyme activity, and transcript abundance from the same material. However, in the rapid harvest (with less than 1 s between cutting and quenching in liquid nitrogen), very few significant differences were found between metabolite levels in M and BS ((iso)-citric acid and malonic acid were both enriched in BS slice 3–4; FDR<0.05; Fig. S2C at Dryad). In contrast, many metabolites showed significant differences based on leaf age (10 metabolites with FDR<0.05 between slice 3–4 and slice 1–2 in the sub-1 s harvest, and 20 with FDR<0.05 between at least one of the neighboring slices in the 10 s harvest; Fig. S2E and Dataset S1 at Dryad). The observed developmental changes were very similar between the sub-1 s and 10 s harvest; however, there were a few exceptions. One example is phenylalanine, which increased in abundance with leaf age in the fast harvest, but decreased in the slow harvest (Fig. S2C, D at Dryad). Although not statistically significant, the BS *vs* M trend of several metabolites corresponded with expectations. Notably, serine and the other photorespiratory metabolites were higher in the BS, where they are expected to be produced, both in the faster (Fig. 1B) and, to a lesser extent, also in the slower (Fig.S2B at Dryad) harvest. Malate, which presumably moves from M to BS entirely based on a diffusion gradient, tended towards enrichment in the mature M (slices 1–2, 1–3; Fig. 1A and Fig. S2A at Dryad). Further, there is a modest consistency between previous studies measuring distribution of metabolites and that measured here (Fig. 1C). All measured core C_4_ metabolites shift from putative BS towards putative M enrichment between slice 3–4 and 1–2 (Fig. 1A). Such synchronized changes could relate to increasing flux (or changing rate-limiting steps) in the C_4_ cycle. The differences between harvest speeds highlights how labile these metabolites can be, and discrepancies between studies or low enrichment values may simply reflect response to conditions and the readiness with which they pass the plasmodesmata, respectively. Higher confidence in metabolite distribution will require more replicates, and, potentially, more defined conditions.

### Comparison with other separated transcriptomes

#### Quantitative study comparison

**Fig. 2.**
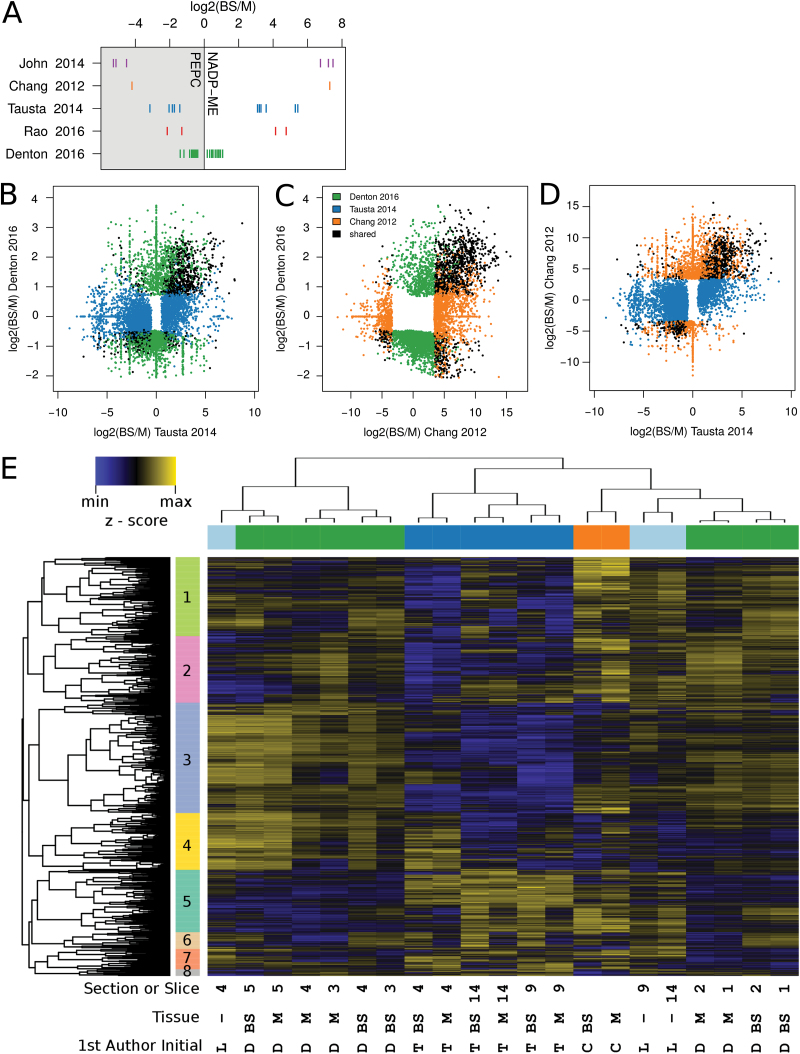
Interstudy comparison. (A) Enrichment of the classic BS (NADP-ME) and M (PEPC) marker genes in each study. (B–D) Log_2_ fold change of genes that were significantly enriched in BS or M in at least one of the paired *Z. mays* studies. (E) Hierarchical clustering of fully normalized log2 (TPM) for *Z. mays* samples, with Pearson and Spearman correlation-based distance for genes and samples, respectively. Genes filtered to those with TPM min>0, max>50. Side colors included to help delineate studies on the *x*-axis and major clusters on the *y*-axis. C, Chang *et al.* (2012); D, this study; L, Li *et al.* (2010); T, Tausta *et al.* (2014).

While this separation method provides high integrity and allowed us to simultaneously measure transcripts, metabolites, and enzyme activities, it comes with its own caveats due to the limited enrichment. As separation studies will likely continue, either in new species or with variations such as separating the husk (Huang and Brutnell, 2016), we evaluated the advantages and disadvantages of different separation methods and their effect on biological results. We compiled a comparative dataset from all existing M/BS specific full RNAseq experiments in monocots. These covered mechanical and enzymatic separation in *Z. mays* (Chang *et al.*, 2012); mechanical separation in *S. viridis* (John *et al.*, 2014); laser micro-dissection in *Z. mays* (Li *et al.*, 2010; Tausta *et al.*, 2014); mechanical micro-dissection in *P. virgatum* (Rao *et al.*, 2016); and the serial filtration performed here (referred to as ‘Denton 2016’ in figures). While the data encompass three origins and two subtypes of C_4_ photosynthesis, and BS and M cell specificity is not expected to match perfectly, previous studies have found substantial conservation even between monocots and dicots (Aubry *et al.*, 2014). Overall, the combination of mechanical BS preparation and enzymatic (Chang *et al.*, 2012) or leaf rolling (John *et al.*, 2014) M separation achieved the highest marker enrichment, followed by the micro-dissection studies (Li *et al.*, 2010; Tausta *et al.*, 2014; Rao *et al.*, 2016), while the method used here, as anticipated from the original report (Stitt and Heldt, 1985), showed the least enrichment (Fig. 2A). Consistent with the lower enrichment, this study showed the lowest statistical power of the various methods with an average of 2100 discoveries (FDR<0.05) per slice, compared with 4030–12 777 discoveries for the other (biological-replicate-including) studies when computed with edgeR. Therefore, a cross contamination aware R-package, contamDE, which includes a factor for the relative tissue enrichment of each replicate, was employed. With contamDE an average of 4479 discoveries (BS-e *vs* M-e FDR<0.05) were made per slice, and this was used for further analysis (see Table S2 at Dryad).

Tissues were matched to achieve a more in-depth comparison between the *Z. mays* studies. For mature tissues, the sample from Chang *et al.* (2012) was most similar to section 14 from Tausta *et al.* (2014) and to slice 2, here, while the youngest section in Tausta *et al.* (2014) was most similar to slice 4, here (Spearman correlation, Fig. S3A at Dryad). The Tausta *et al.* (2014) study was able to detect genes with a lower log fold change (relative to the total log fold change distribution) than either Chang *et al.* (2012), with just one replicate, or this study, with low enrichment. However, examining log fold change indicated the differences between studies ran deeper than statistical power, with many genes significant in one study not enriched or even significantly enriched in the opposite direction in another study (Fig 2B–D).

#### Qualitative study comparison

**Fig. 3.**
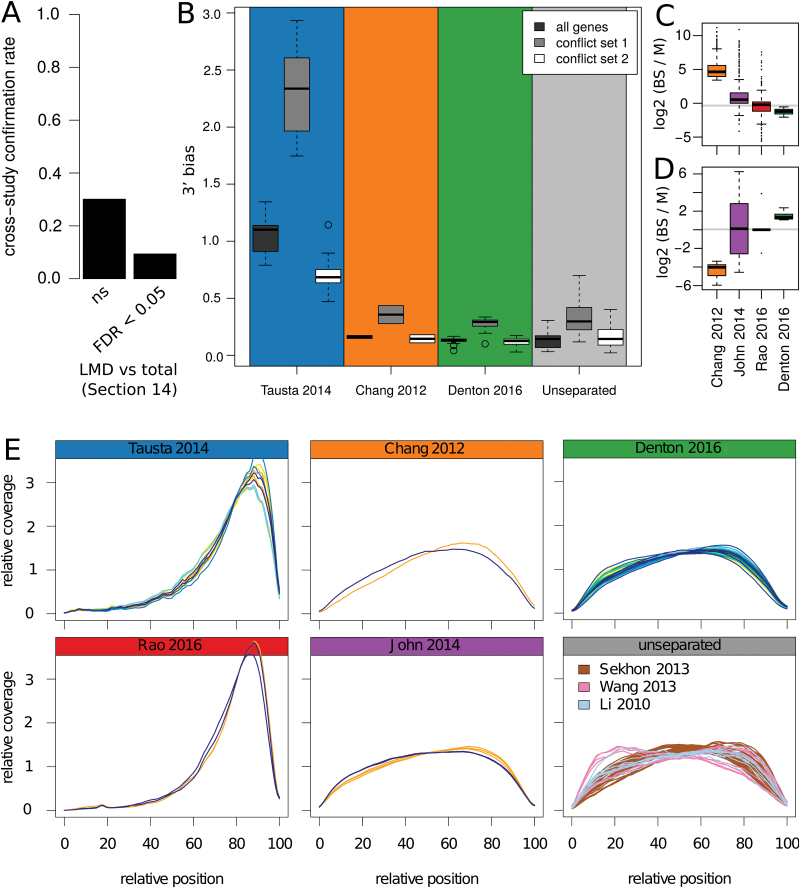
Technical bias. (A) The fraction of significant differences discovered here (slice 2) that were shared with the Tausta *et al.* (2014; section 14) study broken up based on whether these genes were of significantly lower abundance in the laser micro-dissected section 14 compared with whole section 14 (Li *et al.*, 2010; Tausta *et al.*, 2014). (B) The 3′ bias observed in the coverage for the genomic background and the two conflict sets in each *Z. mays* separation study, and all the unseparated samples of Li *et al.* (2010), Wang *et al.* (2013), and Sekhon *et al.* (2013). (C, D) The tissue enrichment of the *Z. mays* genes and the *S. viridis* (John *et al.*, 2014) and *P. virgatum* (Rao *et al.*, 2016) orthologs where the *Z. mays* gene was significantly more abundant in the BS in Chang *et al.* (2012), and the M in slice 2 (this study) (C), or vice versa (D). (E) Transcript coverage by study. For all BS and M separation studies, blue represents BS, yellow represents M, and tissue maturity increases from light to dark. Green represents I-e in this study (Denton 2016).

For a more qualitative look at the differences between studies we performed a hierarchical clustering of samples from this study, those from Chang *et al.* (2012) and Tausta *et al.* (2014), and the unseparated sections from Li *et al.* (2010) that corresponded to Tausta *et al.* (2014). The samples clustered primarily by study, followed by leaf age and then M and BS, with some mixing (Fig. 2E). Between-study differences could in theory come from growth conditions and plant age, from differences in separation method or from a combination thereof, and all studies but Li *et al.* (2010) and Tausta *et al.* (2014) used distinct growth and harvest conditions (see Table S3 at Dryad). Notably, the unseparated sections from Li *et al.* (2010), which were grown comparably to those from Tausta *et al.* (2014) clustered not with the associated leaf sections of Tausta *et al.* (2014), but with the respective older or younger serial filtration data here, indicating a substantial role of separation method in clustering. Indeed, one of the gene clusters (3) was primarily expressed at a lower level across the laser micro-dissection (Tausta *et al.*, 2014) samples compared with all the other samples (including Li *et al.*, 2010). RNA is known for its degradability under procedures like laser micro-dissection, and Li *et al.* (2010) clearly reported the 3′ bias in the laser micro-dissection section 14, but did not at that time have the comparative studies to evaluate how this would globally affect the results. A list of genes most dramatically affected by laser micro-dissection was obtained by looking for genes with significantly different abundance between unseparated (Li *et al.*, 2010) and the laser micro-dissection separated section 14 (Li *et al.*, 2010; Tausta *et al.*, 2014). The majority (3298 of 3362) of the differentially regulated genes were downregulated in the laser micro-dissection samples. These laser micro-dissection ‘downregulated’ genes were depleted in BS *vs* M, differences shared with this study (Fig. 3A; Fisher’s exact test, *P*<0.001). Further, these genes showed several functional enrichments (MapMan categories), including major categories such as transport and signaling; and minor categories such as minor CHO metabolism.callose, GARP G2-like transcription factor family and Class XI Myosin (Dataset S2 at Dryad). Finally, the strong 3′ bias resulted in a low diversity library compared with the other studies (see Fig. S3B, C at Dryad).

Considering the effect degraded RNA can have, we evaluated the 3′ bias across studies to see how the other separation methods compared. The three prime bias was highest in the laser (Tausta *et al.*, 2014) and mechanical (Rao *et al.*, 2016) micro-dissection studies; however, it was present to various degrees in at least some samples of the other separation studies and in multiple other *Z. mays* studies without separation (Li *et al.*, 2010; Sekhon *et al.*, 2013; Wang *et al.*, 2013; Fig. 3C). Notably, both studies that used distinct methods for isolation of BS strands and M cells (John *et al.*, 2014; Chang *et al.*, 2012) showed minor 3′ bias, but each M sample showed more than its corresponding BS sample (Fig. 3C). The 3′ bias was not spread evenly across all genes, but was higher in the 199 genes where Chang *et al.* (2012) and slice 2 (this study) were significantly, but oppositely, enriched in the BS and M, respectively (see Fig. S4 at Dryad). Overall mild increases in 3′ bias between samples are prominent in these 199 genes and their orthologs, notably including the M samples in Chang *et al.* (2012) and Tausta *et al.* (2014) and one BS replicate from this study. The orthologs of these 199 genes, measured by John *et al.* (2014) mostly (138 of 184; 75%) were enriched in the same direction as Chang *et al.* (2012), while those measured by Rao *et al.* (2016) mostly (101 of 152; 66%) agreed with this study (Fig. 3C). In contrast, neither cross-species comparison showed a notable BS or M bias in orthologs of the opposite gene set—the 14 genes where Chang *et al.* (2012) and slice 2 (this study) were significantly enriched in the M and BS, respectively (Fig. 3D). In summary, despite evolutionary distance between *Z. mays* and *S. viridis*, the studies with higher, degradation-marking 3′ bias in the M than BS (Chang *et al.*, 2012; John *et al.*, 2014) share a set of ‘BS enriched’ genes that conflict with the M enrichment seen in *Z. mays* (this study) and *P. virgatum* (Rao *et al.*, 2016).

**Fig. 4.**
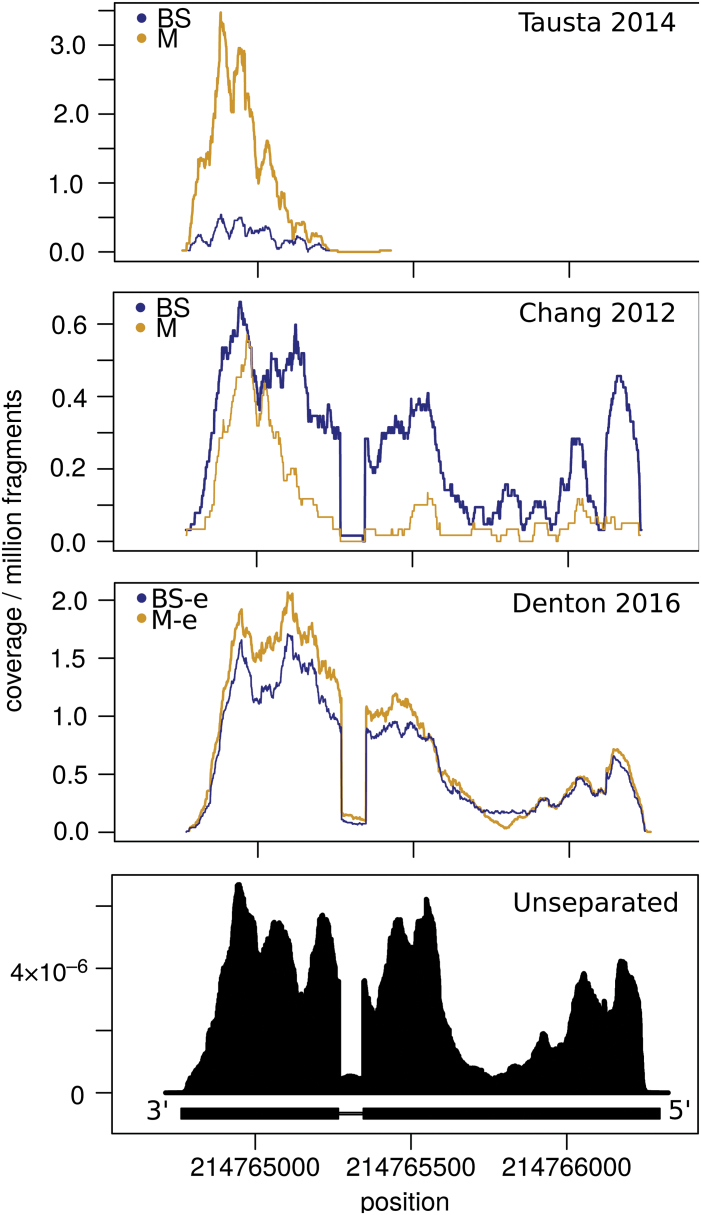
Coverage of example gene PPCK. Read depth across genomic region of PPCK (GRMZM2G178074; which is in conflict set 1) in the various *Z. mays* separation studies, and in the unseparated samples of Li *et al.* (2010), Wang *et al.* (2013), and Sekhon *et al.* (2013).

To determine if different RNA quality and 3′ bias relate to some of the discrepancies between the *Z. mays* studies, we quantified the level of 3′ bias on genes in two different conflict sets—conflict set 1: BS specific in Chang *et al.* (2012) or Tausta *et al.* (2014) and M specific in the comparable tissue here, or BS (Chang *et al.*, 2012) and M (Tausta *et al.*, 2014, section 14); conflict set 2: as conflict set 1 but with BS and M switched). This showed that conflict set 1 had the most 3′ bias across all studies while conflict set 2 had the same or even less bias than the whole gene set (Fig. 3B). One of the genes in ‘conflict set 1’ is related to the C_4_ cycle, namely phosphoenolpyruvate carboxylase kinase (PPCK; GRMZM2G178074), which regulates PEPC in the M (Vidal and Chollet, 1997). The coverage across the PPCK locus shows a mild 3′ bias in unseparated studies and in both BS and M samples here, with higher coverage in the M (Fig. 4). In the laser micro-dissection study, there is a strong 3′ bias in both samples, with more remaining coverage in the M sample, while in the Chang *et al.* (2012) sample, there is a mild 3′ bias in the BS sample, but a strong 3′ bias in the M sample, causing PPCK to appear higher in the BS based on total read count. While not all genes in ‘conflict set 1’ looked like this (e.g. many had a very strong 3′ bias across every sample and study; not shown), other similar examples were not hard to find (see Fig. S5 at Dryad). Further, components of ‘conflict set 1’ were enriched in several MapMan categories. These included three transcription factor sub-categories (PHD finger, pseudo ARR, and putative), and minor CHO metabolism.callose in genes BS specific in Chang *et al.* (2012), and M specific in slice 2 of this study (Dataset S2 at Dryad).

Another likely artifact of the separation method is the residual contamination with non-M and non-BS tissue types. The mechanical separation methods are expected to co-purify the vascular bundle with the BS cells, while the serial grinding and filtration used here presumably includes all cell types in at least one of the enrichment fractions. To confirm and quantify these expectations would require unambiguous markers that were known to, for instance, be highly specific to the vascular tissues and absent from M or BS. In the absence of fully characterized markers in *Z. mays*, we tested a variety of candidates, largely known from other species.

**Fig. 5.**
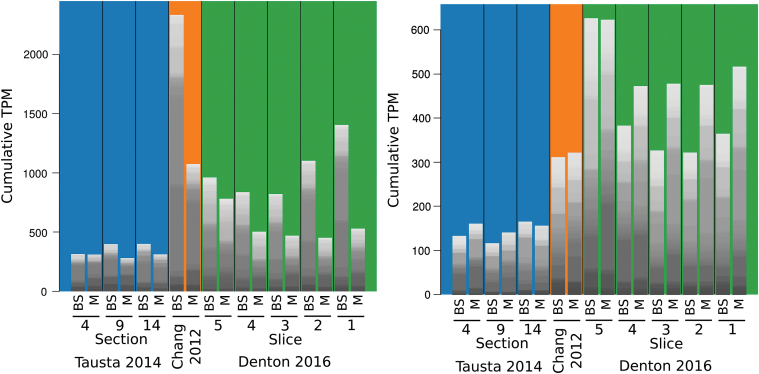
Co-purification of additional tissues. Fully normalized abundance of genes that included the word ‘phloem’ (A) or ‘epidermal’ (B) in their MapMan description.

Putative vascular markers were initially selected from the literature based on functions expected to be highly vascular specific. Enzymes associated with lignification of proto-xylem elements (LAC17) were more abundant in the BS base sample (Fig. S6A at Dryad; FDR<0.05 for three of the four expressed). Similarly, homologs to Arabidopsis XYLEM CYSTEIN PROTEASE (XCP) 1 (GRMZM2G066326) and 2 (GRMZM2G367701), involved in programmed cell death in the xylem, were higher in the BS base sample (Fig. S6B at Dryad; FDR<0.001 for all three). These markers, however, were not expressed in older tissue and thus could not be used for interstudy comparisons. SUCROSE TRANSPORTER 2 (SUT2), frequently used as a companion cell marker in Arabidopsis (AT2G02860; Meyer *et al.*, 2000), has five homologs in *Z. mays*, for which the cumulative expression was enriched in the BS across all *Z. mays* studies (see Fig. S6C at Dryad). A study on phloem transported RNAs in Arabidopsis (Deeken *et al.*, 2008) provided a larger list of potential vascular markers; however, the cumulative expression was again higher in the BS across studies (Fig. S6D at Dryad). We further examined sets of genes that included ‘phloem’ (Fig. 5A), ‘xylem’ (Fig. S6E at Dryad), or ‘vascular’ (Fig. S6F at Dryad) in their descriptions. Cumulative expression of these keyword gene sets was largely higher in the BS across studies; however, for ‘phloem’ and ‘vascular’ genes, BS enrichment in the laser micro-dissected samples was less than BS enrichment of these genes in the mechanical separation studies.

We further evaluated the distribution of putative epidermal markers. A previous study using laser micro-dissection to separate epidermal and M tissues identified two epidermal specific genes in *Z. mays* (Javelle *et al.*, 2010). The more highly expressed of these, GRMZM2G345700, was consistently higher in the M samples (Fig. S6G at Dryad; FDR<0.05 in six of nine comparisons), while the less highly expressed GRMZM2G387360 was not significantly enriched. A broader look at all genes including the words ‘epidermal’ in their descriptions (Fig. 5B) showed higher cumulative expression in the M in most comparisons, while the most substantial M enrichment appeared to be in this study (Fig. 5B). It is hard to draw a firm conclusion in the absence of unambiguous markers, as expression patterns in epidermal cells may be more similar to M than BS, and vice versa for vascular expression. However, both expectation and a view on the broader patterns support co-purification of vascular tissues with the mechanical BS purification methods, co-purification of epidermal tissue with the M in the serial filtration method used here, and generally less co-purification using laser micro-dissection.

### The strengths of interstudy comparison

**Fig. 6.**
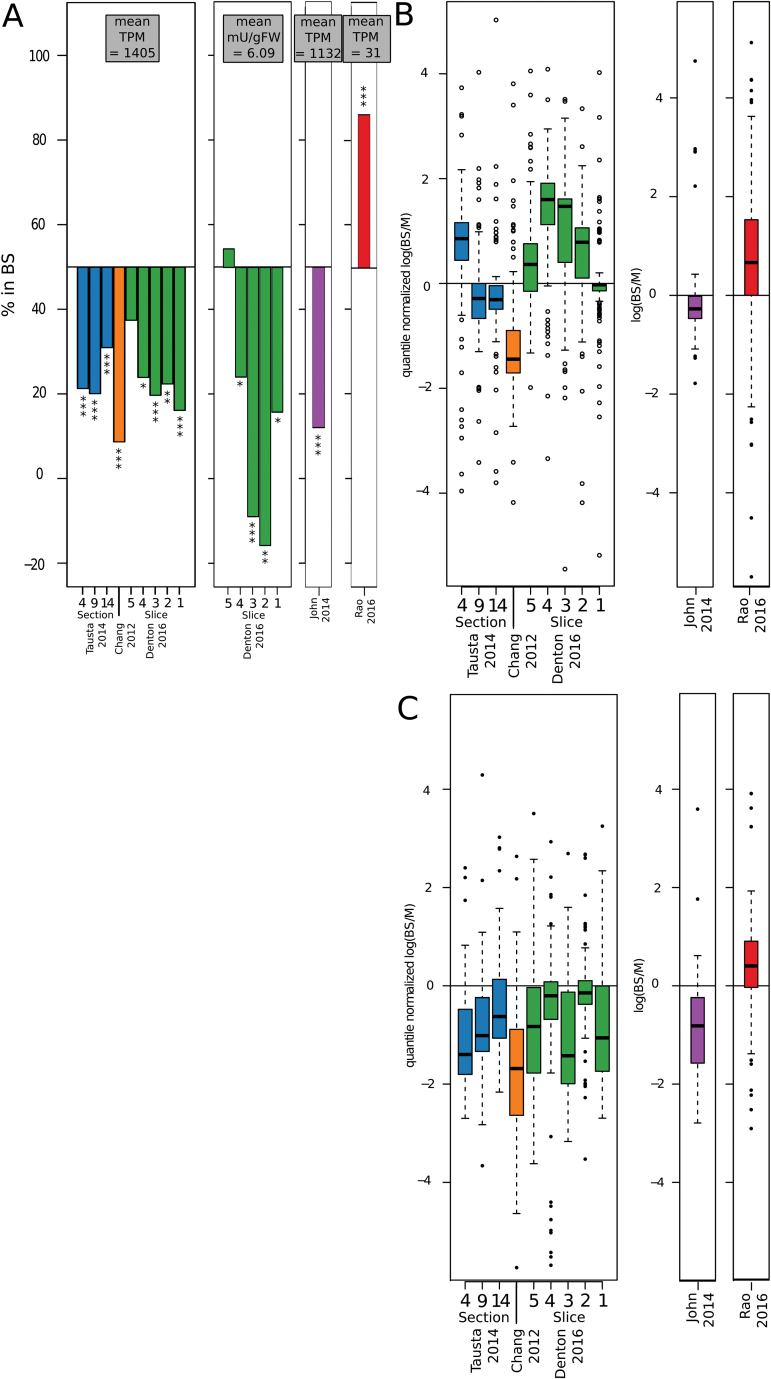
Biological insights drawn from interstudy comparison. (A) Tissue enrichment of AspAT of transcripts in maize (left), enzyme activity in *Z. mays* (mid-left), transcripts in *S. viridis* (mid-right) and transcripts in *P. virgatum* (right). (B, C) Tissue enrichment of transcripts in the MapMan functional category for 60S ribosomal protein (B) and photosystem II (C) in *Z. mays* (left), *S. italica* (middle), and *P. virgatum* (right). In (A) asterisks denote significance of FDR for transcripts and *P*-values for enzyme activity (*P<0.05, **P<0.01, ***P<0.001).

Multiple study comparisons allow for confidence in results that would seem dubious alone. In this study, aspartate aminotransferase stood out as having transcript enrichment in M cells (Fig. 6A) that was contrary to the expected even distribution between cell types in the current *Z. mays* C_4_ model (Furbank, 2011; Pick *et al.*, 2011). Comparison with the other datasets confirmed the same pattern in all NADP-ME studies (*Z. mays* and *S. viridis*). Previous studies (Chang *et al.*, 2012; Tausta *et al.*, 2014) have mentioned a low-expression BS specific AspAT paralog, or the detection of AspAT in both BS and M proteomic studies as balancing explanations. However, in both transcriptomics and proteomics (Friso *et al.*, 2010; Majeran *et al.*, 2010) the total abundance is much higher in the M. This is further supported by the high M specificity of the AspAT enzyme activity (Fig. 6A). A similar but less pronounced pattern in transcripts could be found for alanine aminotransferase (see Fig. S7A at Dryad).

Consistent BS or M enrichment as the leaf develops helps increase confidence, both as a repeat observation and as a simple explanation consistent with the gradual nature of changes in transcript abundance during leaf development (Pick *et al.*, 2011). On the flip side, however, it seems less likely that a gene changed from BS specific to M specific or vice versa during development. We used the interstudy comparison to evaluate the reliability of observed switches in enrichment across leaf development. As expected, genes that were significantly enriched in M-then-BS or BS-then-M in sections 4 and 14 of Tausta *et al.* (2014) were much less likely to find cross-study support (same enrichment direction in slice 4 and 2 of this study) than their M-then-M or BS-then-BS enriched counterparts (19% *vs* 78%, Fisher’s exact test *P*<0.001). However, the 48 genes that were significantly enriched in the BS in section 4 (Tausta *et al.*, 2014) and in the M in section 14 (Tausta *et al.*, 2014) with support from this study showed enrichment in the functional category ‘protein.synthesis.ribosomal protein.eukaryotic.60S subunit’ and all parental categories there of (Dataset S2 at Dryad). Further investigation showed that both the 60S and 40S ribosomal subunits have a clear pattern with strong BS enrichment in young but entirely unsheathed tissue (section 4, Tausta *et al.*, 2014; slice 4, here). As the leaf develops the strong BS enrichment fades, and even switches to a mild M enrichment (Fig. 6B and Fig. S7B at Dryad). To determine if the mature M enrichment could be related to supporting the high turnover of photosystem II components, we included the data for *S. viridis* (John *et al.*, 2014) and *P. virgatum* (Rao *et al.*, 2016) in the analysis. Notably the 60S and 40S ribosomal subunits showed M enrichment in *S. viridis* (Fig. 6B and Fig. S7B at Dryad), in which photosystem II, like in *Z. mays*, is primarily localized to the M (Fig. 6C). In contrast, these subunits showed BS enrichment in *P. virgatum* (Fig. 6B and Fig. S7B at Dryad), in which photosystem II is not primarily localized to the M (Fig. 6C).

### Data accessibility and visualization

**Fig. 7.**
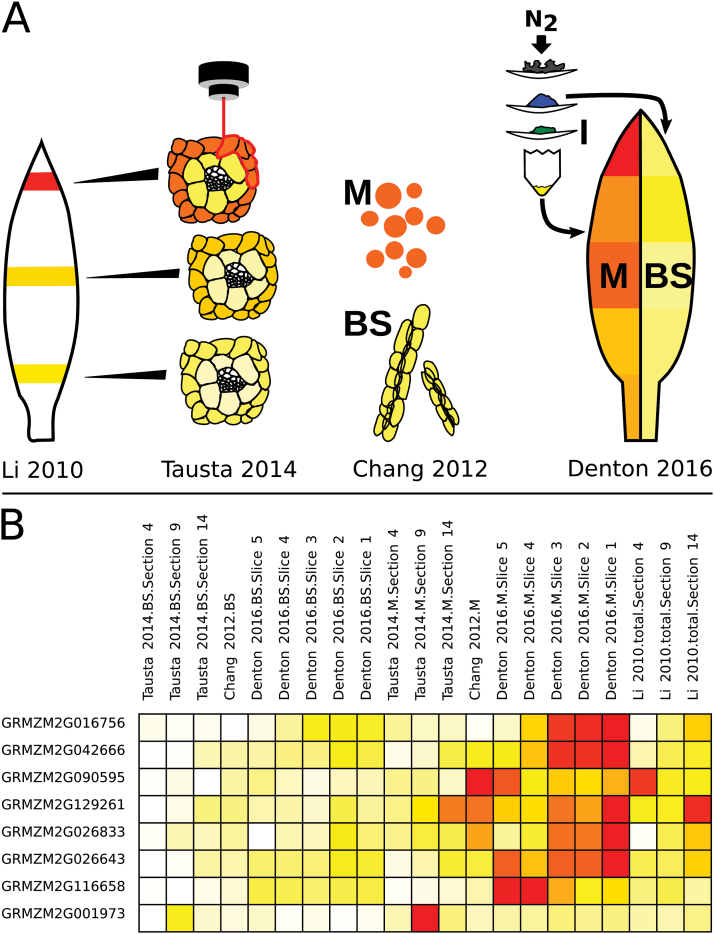
Web visualization resource. (A) Comparative BS and M separation targeted graphical heatmap view of example gene (GRMZM2G129261). (B) Example gene set visualization of highest confidence M transcription factors.

To facilitate public comparison of these transcriptomes, we are providing (i) a *Z. mays* gene browser with gene-specific or gene-group visualization of the data from BS/M separation studies in *Z. mays*; and (ii) all the data analysed in this study (including non-*Z. mays* BS *vs* M comparisons, and unseparated *Z. mays* studies) in tabular format (Dataset S3 and S4 at Dryad). The *Z. mays* gene browser aims to facilitate comparison and critical evaluation of the similarities and differences between these studies. To this end, the graphics include the separation method in the display and necessary contextual data (e.g. unseparated samples from Li *et al.* (2010) corresponding to the laser micro-dissection samples from Tausta *et al.* (2014), and 3′ bias (Fig. 7). Further, the browser includes several pre-loaded gene sets to help users compare studies (Fig. 7B). These sets include, for example ‘conflict set 1’ described above. Further gene sets include three gradations of highly supported M or BS specific genes across studies (735, 365, and 126 significant differences; shared between 7+, 8+, or all 9 of the comparisons, respectively), and highly supported M or BS specific transcription factors (52 significant differences shared between 7+ comparisons), and transcription factors of special interest in immature tissue (36 significant differences in two of the three youngest comparisons (Tausta *et al.*, 2014 section 4, and slice 4 and 5, here) and higher in foliar than husk primordia in Wang *et al.* (2013). Full lists and descriptions are provided in Dataset S5 at Dryad and with the visualization tool at http://www.plant-biochemistry.hhu.de/resources.html.

## Discussion

Despite the variety of BS and M separation methods used and increasing number of studies, no method presents itself as a clear best option. Rather, the various methods come with advantages and disadvantages, which should be considered both when planning the experiment and evaluating the data.

The only fast methods for metabolite extraction are leaf rolling (Leegood, 1985) for M compared with whole tissue, and grinding and serial filtration on liquid nitrogen as performed here (Stitt and Heldt, 1985). Only a handful of metabolites measured in these studies overlap, and the correlation between studies is modest. Elements of this study might help clarify why and plan the next experiment. First, there were very substantial differences between the <1 s harvest and the 10 s harvest and between the different leaf slices. This highlights that the dynamics of abundance of metabolites make them extremely sensitive to both conditions and harvest methods. Considering the dominance of age, conditions and method over BS *vs* M differences in the clustering of RNAseq data, it is perhaps unsurprising that the even more labile metabolites continue to pose challenges. Similarly, the low absolute enrichment of this method and the Leegood (1985) method decreases the signal to noise ratio, particularly making identification of low log fold changes between cell types difficult (as seen in the RNAseq). This is likely exacerbated by the division of some metabolites, such as aspartate and malate, into active and inactive pools. These inactive pools can be substantial, accounting for about 60% and 80% of the total aspartate and malate, respectively, in the grass *Chloris gayana* (Hatch, 1979). In contrast, the high density of plasmodesmata between M and BS cells in C_4_ plants supports diffusion of C_4_-cycle metabolites at the rate of carbon fixation (Laisk and Edwards, 2000); it is thus implausible that any cytoplasmic metabolite could build up enrichment levels comparable to transcripts and enzymes. Therefore a study prioritizing understanding metabolic differences between BS and M cells should err on the side of a few more replicates than the five that is the ‘industry standard’ for metabolic studies (Sumner *et al.*, 2007). Similarly, sequencing a few more than the typical two to three replicates for RNAseq may help compensate for the lower sensitivity of this method.

For any study not targeting metabolites, the higher purity achieved by any of the other methods over the method here has an obvious allure; however, the biases associated with lower quality RNA must be accounted for. As shown here and reported previously (Romero *et al.*, 2014) RNA does not degrade at consistent rates, but rather some RNA molecules, often including transcription factors (Yang *et al.*, 2003), are much more sensitive to degradation. These degradation-sensitive genes are numerous (12.5% of detectable genes showed significantly lower abundance after laser micro-dissection; Li *et al.*, 2010; Tausta *et al.*, 2014). Further, shared genes with bias in Chang *et al.* (2012) and John *et al.* (2014) indicate degradation sensitivity is conserved across species and can masquerade as conserved tissue specificity. For the above reasons, care must be taken not to intermingle any biological signal sensitive to degradation and the biological signal between samples. For instance, the two callose synthases that Chang *et al.* (2012) discussed as being BS specific (GRMZM2G553532 and GRMZM2G004087) appear to be very sensitive to degradation as they are both among the genes significantly less abundant after laser micro-dissection, and one, GRMZM2G553532, is in the conflict set 1 list with strong 3′ bias. This raises the worrisome question of whether this is a case of differential expression, or differential degradation. Future studies may be able to circumvent such problems by including a third and unseparated sample that can be used to detect genes particularly affected by degradation—much as we’ve been using the unseparated section 14 from Li *et al.* (2010) as the context for the Tausta *et al.* (2014) separated section. This method has been employed by a recent study using SuperSage on mechanically separated BS and M protoplast in Sorghum (Döring *et al.*, 2016).

A more ideal solution is of course to avoid mixing biological and technical signals by handling RNA in a fashion that preserves RNA quality or at least results in the same amount of degradation in the M and BS samples. Quality control must be performed carefully as a study using the same separation technique as John *et al.* (2014) for qPCR in sorghum achieved very comparable bioanalyser traces for their mechanical BS purified and their leaf-rolled M samples (Covshoff *et al.*, 2013). Indeed, this method was specifically employed for its speed and lack of stress response compared with M protoplast isolation, but still showed distinctly higher 3′ bias in M than BS (John *et al.*, 2014). Thus if a distinct method is to be used for M and BS purification, equivalent RNA needs to be confirmed for the particular species and particular researcher, and not simply assumed based on literature.

While the micro-dissection studies had the strongest overall 3′ bias, there was equivalent bias in the M and BS samples. This resulted in false negatives and lower library complexity, but had no clear link to false positives. In the microdissection studies, an alternative explanation for the 3′ bias is the synthesis of the first strand cDNA using an Arcturus Ribo Amp HS kit, which has been shown to induce a strong 3′ bias in housekeeping genes (Clément-Ziza *et al.*, 2009). This does not, however, nullify the substantial differences and loss of transcript detection seen between the laser micro-dissected (Li *et al.*, 2010; Tausta *et al.*, 2014) and the unseparated samples (Li *et al.*, 2010). There is ongoing research in improving laser-micro-dissection techniques in plants (Ludwig and Hochholdinger, 2014). We recommend that while techniques remain uncertain, researchers invest the necessary time and money in quality control steps and unseparated controls to assure that the bias that is there is traceable.

Use of a different bioinformatics workflow may make a small difference in the measured abundance of genes with a strong 3′ bias, but a perfect solution is not yet available, particularly as tools are not optimized for this. Small additions to a typical workflow, such as flagging discrepancy in 3′ bias between groups (e.g. Chang *et al.*, (2012)’s samples in Fig. 4), could help avoid erroneous conclusions.

Where one study has weaknesses, interstudy comparison can provide a helpful additional opinion. The completion of a third *Z. mays* M and BS separation RNAseq study with a complementary technique here continued to yield new biological results. Particularly in areas where results may seem dubious, consensus between several studies (with different techniques or information gathered) is required to gain confidence. An example of this is AspAT’s consistent M localization, which while previously noted (Chang *et al.*, 2012; Tausta *et al.*, 2014), was not taken seriously without the supporting enzyme activity data. It may have a simple explanation such as a higher substrate to product ratio in the BS requiring less enzyme, or a more complex one such as an aspartate pool in the M simply adding stability to CO_2_ fixation should diffusion or decarboxylation of malate become temporarily limiting. Either way, this warrants further investigation. Similarly, the switch from BS to M specificity of ribosomal proteins is much easier to trust when identified in two independent studies. Differentiation of veins and the associated BS cells precedes that of the M, and signals from the BS are necessary for M differentiation in Arapidopsis (Kinsman and Pyke, 1998; Lundquist *et al.*, 2014), and the C_4_ dicot *Gynandropsis gynandra* shows the same developmental trajectory (Külahoglu *et al.*, 2014). Therefore, we hypothesize the initial BS enrichment in protein synthesis may reflect faster differentiation and photosynthetic ramp-up in the BS cells. As the photosynthetic rate increases along the developing leaf (Pick *et al.*, 2011), the shifting of the protein synthesis towards the M likely supports the high turnover of photosystem II subunits (Rokka *et al.*, 2005). Considering that photosynthesis-related proteins make up over half of mature leaf protein (Friso *et al.*, 2010; Majeran *et al.*, 2010), the distribution of protein synthesis in mature leaf may reflect the balance between the demand from synthesizing photosystem II (when in the M) and synthesizing Rubisco and the other BS-specific CBBC enzymes.

Altogether, the separation technique of choice depends upon the research question. In many cases the weaknesses of one study are compensated for by the strengths of another, particularly when biases are characterized and taken into consideration. This work provides a visual access tool summarizing this study and Li *et al.* (2010), Chang *et al.* (2012) and Tausta *et al.* (2014), tables of all data looked at here (above and Wang *et al.*, 2013; Sekhon *et al.*, 2013; John *et al.*, 2014; Rao *et al.*, 2016), and highlights biological observations drawn from the sum of many studies.

## Data deposition

The following data are available at Dryad Digital Repository http://dx.doi.org/10.5061/dryad.tf6q6.

Datasets S1. Enzyme activity and metabolite abundance.

Datasets S2. Functional enrichments.

Datasets S3. Compiled RNAseq data.

Datasets S4. By gene 3′ bias.

Datasets S5. Gene sets of interest.

Fig. S1. Setup and confirmation of separation method.

Fig. S2. Metabolite enrichment.

Fig. S3. Contextual data for interstudy comparison.

Fig. S4. Coverage of BS (Chang *et al.*, 2012) *vs* M (this study) conflict genes.

Fig. S5. Example read coverage.

Fig. S6. Co-purification of additional tissues.

Fig. S7. AlaAT and 40S ribosome distributions.

Table S1. Bioinformatics parameters.

Table S2. Counting significant differences.

Table S3. Harvest and growth conditions.

## Competing interests

The authors declare that they have no competing financial interests.

## Author contributions

AKD, AB and APMW designed the study. AKD performed wetlab measurements, computational, statistical and general data analysis and wrote the manuscript. JM developed the visualization tool, performed general data analysis, and assisted in wetlab measurements and writing the manuscript. CK, MJL, AB, and APMW contributed to data analysis, interpretation of the results, and writing the manuscript.
