# Corrigendum: Freeze-quenched maize mesophyll and bundle sheath separation uncovers bias in previous tissue-specific RNA-Seq data

**DOI:** 10.1093/jxb/ery196

**Published:** 2018-06-05

**Authors:** Alisandra K Denton, Janina Maß, Canan Külahoglu, Martin J Lercher, Andrea Bräutigam, Andreas P M Weber

**Affiliations:** 1 Institute of Plant Biochemistry, Cluster of Excellence on Plant Sciences (CEPLAS), iGRAD-Plant Program, Heinrich-Heine-University, Düsseldorf, Germany; 2 Institute of Informatics, Cluster of Excellence on Plant Sciences (CEPLAS), iGRAD-Plant Program, Heinrich-Heine University, Düsseldorf, Germany; 3 Network Analysis and Modeling Group, IPK Gatersleben, Corrensstrasse, Stadt Seeland, Germany

*Journal of Experimental Botany,* Vol. 68, No. 2 pp. 147–160, 2017 doi: 10.1093/jxb/erw463

The original version of this manuscript wrongly cited a paper. The citation was as follows:

Stitt M, Heldt H W. Control of Photosynthetic Sucrose Synthesis by Fructose 2,6-Bisphosphate. VI. Regulation of the Cytosolic Fructose 1,6-Bisphosphatase in Spinach Leaves by an Interaction between Metabolic Intermediates and Fructose 2,6-Bisphosphate. Planta, 1985, 164(2):599–608.

The manuscript has now been updated to include the correct citation. The correct citation is as follows:

Stitt M, Heldt H. 1985. Control of photosynthetic sucrose synthesis by fructose-2, 6-bisphosphate. Planta. 164(2):179–88

The authors would like to apologise for this error.

